# Mitochondrial genomes of the hoverflies *Episyrphus balteatus* and *Eupeodes corollae* (Diptera: Syrphidae), with a phylogenetic analysis of Muscomorpha

**DOI:** 10.1038/srep44300

**Published:** 2017-03-09

**Authors:** De-qiang Pu, Hong-ling Liu, Yi-yun Gong, Pei-cheng Ji, Yue-jian Li, Fang-sheng Mou, Shu-jun Wei

**Affiliations:** 1 Industrial Crop Research Institute, Sichuan Academy of Agricultural Sciences, Chengdu 610300, China; 2 Institute of Plant Protection, Sichuan Academy of Agricultural Sciences, Chengdu 610066, China; 3 Institute of Horticulture Research, Sichuan Academy of Agricultural Sciences, Chengdu 610066, China; 4 Institute of Plant and Environmental Protection, Beijing Academy of Agriculture and Forestry Sciences, Beijing 100097, China

## Abstract

The hoverflies *Episyrphus balteatus* and *Eupeodes corollae* (Diptera: Muscomorpha: Syrphidae) are important natural aphid predators. We obtained mitochondrial genome sequences from these two species using methods of PCR amplification and sequencing. The complete *Episyrphus* mitochondrial genome is 16,175 bp long while the incomplete one of *Eupeodes* is 15,326 bp long. All 37 typical mitochondrial genes are present in both species and arranged in ancestral positions and directions. The two mitochondrial genomes showed a biased A/T usage versus G/C. The *cox1, cox2, cox3, cob* and *nad1* showed relatively low level of nucleotide diversity among protein-coding genes, while the *trnM* was the most conserved one without any nucleotide variation in stem regions within Muscomorpha. Phylogenetic relationships among the major lineages of Muscomorpha were reconstructed using a complete set of mitochondrial genes. Bayesian and maximum likelihood analyses generated congruent topologies. Our results supported the monophyly of five species within the Syrphidae (Syrphoidea). The Platypezoidea was sister to all other species of Muscomorpha in our phylogeny. Our study demonstrated the power of the complete mitochondrial gene set for phylogenetic analysis in Muscomorpha.

The mitochondrion is a fundamental eukaryotic organelle. Energy production via oxidative phosphorylation is its most-studied function, though it also functions in apoptosis and cell aging^1^,^2^. A typical animal mitochondrial genome is a circular DNA molecule, approximately 16 kb long, with a relatively conserved gene content, usually containing 37 genes: 13 protein-coding genes (PCGs), two ribosomal RNA (rRNA) genes, and 22 transfer RNA (tRNA) genes, plus an A + T-rich region^3^,^4^. Compared with nuclear genomes, animal mitochondrial genomes are characterized by several distinct features, including cellular abundance, small genome size, conserved gene content and organization, lack of extensive recombination, maternal inheritance, and a high nucleotide substitution rate^2^,^5^,^6^. In particular, mitochondrial genomes can provide higher phylogenetic resolution than the shorter sequences of individual genes. Consequently, this small molecule has been widely used for phylogenetic analyses in many groups^2^,^7^,^8^,^9^,^10^,^11^,^12^.

Muscomorpha (Diptera: Brachycera), an infraorder of Brachycera, is a large and diverse group of flies, containing the bulk of the Brachycera. It includes a number of the most common flies, such as the housefly, the fruit fly, and the blowfly. The Muscomorpha can be separated into two sections, the Aschiza and the Schizophora^9^,^13^,^14^,^15^. Two large superfamilies, Platypezoidea and Syrphoidea were traditionally included in Aschiza. Phoridae and Syrphidae are the two largest families within these superfamilies, respectively. The Schizophora contain the majority of family level diversity among dipterans, and represent a recent rapid radiation of lineages^14^,^16^. Schizophora can be divided into two subsections, the Acalyptratae and Calyptratae, commonly referred to as acalyptrate muscoids and calyptrate muscoids, respectively^15^.

Hoverflies, also called flower flies, compose the insect family Syrphidae. This family contains almost 6,000 described species in 200 genera, and is nearly worldwide in distribution^17^,^18^. As the common names suggest, these flies are often seen hovering or nectaring at flowers. In contrast to the fairly uniform flower-feeding habits of adult syrphids, the larvae eat a wide range of foods. Traditionally, the Syrphidae has been divided into three subfamilies, the Eristalinae, Microdontinae, and Syrphinae. Larvae of the subfamily Eristalinae are saprophagous in dead wood, eating decaying plant and animal matter in soil, ponds, or streams^19^,^20^; whereas Microdontinae larvae are inquilines in ants’ nests^21^; and Syrphinae larvae are insectivores, preying on aphids, thrips, and other plant-sucking insects^17^,^22^,^23^. This three subfamily system (Microdontinae, Eristalinae, and Syrphinae) was adopted for Syrphidae more than 25 years ago^18^,^23^.

The marmalade hoverfly *Episyrphus balteatus* (Syrphidae) is a relatively small hoverfly (9–12 mm), widespread throughout the Palearctic ecozone, endemic to Europe, North Asia, and North Africa. Its color patterns may appear wasp-like to animals, such as birds, protecting it from predation^24^. Often exhibiting dense migratory swarm behavior, this, and the resemblance to wasps, may panic unaware people^25^. Its feeding habit is rare among adult flies, as it is capable of crushing pollen grains as a food source^26^. *Eupeodes corollae* (Syrphidae) is another common hoverfly species. The adults are often migratory with a worldwide distribution. It has been tested as a biological control agent for aphids and scale insects in greenhouses. However, the larval flies preferred the fruit in the experiment, consuming more fruit than aphids^27^.

As of June 2016, three Syrphidae mitochondrial genome sequences were deposited into GenBank: *Ocyptamus sativus, Simosyrphus grandicornis* and an unknown genus sp. Syrphidae (accession KM244713)^9^,^16^,^28^. Here, we sequenced the mitochondrial genomes from *Episyrphus* and *Eupeodes*, representing the first mitochondrial genomes reported from the genera *Episyrphus* and *Eupeodes*. Furthermore, we compared genome features and investigated phylogenetic relationships within Muscomorpha using complete mitochondrial genomes from GenBank along with our two newly sequenced mitochondrial genomes (Table 1).

## Results and Discussion

### General features of the newly sequenced mitochondrial genomes

The complete *Episyrphus* mitochondrial genome sequence is 16,175 bp long (GenBank accession KU351241) (Table 2). The partial mitochondrial genome of *Eupeodes* mitochondrial genome sequence is 15,326 bp long (GenBank accession KU379658) (Table 3). Particularly A + T-rich region was failed to generate reliable sequence data in both species.

No gene rearrangement was observed in our analyses: (1) as compared with the putative ancestral insect arrangement^29^, (2) as in all sequenced dipteran species^11^, (3) as with the 23 genes encoded on the majority strand (J-strand), and (4) as with the 14 genes encoded on the minority strand (N-strand).

Each of the 37 typical mitochondrial genes is present in both species. The mitochondrial genome of *Episyrphus* has 255 bp of intergenic nucleotides, in 22 different locations, with intergenic spacer lengths ranging from 1 to 60 bp. Seven pairs of genes overlap each other, with overlap lengths ranging from 1 to 7 bp. Eight pairs of genes directly adjacent one another including the pairs of *rrnL*-*trnV* and *trnV*-*rrnS*. The mitochondrial genome of *Eupeodes* has 230 bp of intergenic nucleotides, in 19 locations, with intergenic spacer lengths from 2 to 47 bp. Nine pairs of genes overlap each other, with overlap lengths ranging from 1 to 7 bp. Nine pairs of genes directly adjacent one another including the pairs of *rrnL*-*trnV* and *trnV*-*rrnS*. In both species, the longest intergenic spacer was located between *trnK* and *trnD*, followed by the one located between *trnE* and *trnF*. The longest overlapping regions were located between *atp8* and *atp6, nad4* and *nad4l*. The intergenic and overlapping region of these two species are similar to most other insect mitochondrial genomes with no gene rearrangement occurred, compared with those with frequent gene rearrangement^30^.

### Base composition

Three parameters, the AT and GC asymmetries, known as AT-skew and GC-skew, and A + T content, are often used to characterize the nucleotide-compositional behavior of mitochondrial genomes^31^,^32^. The *Episyrphus* and *Eupeodes* mitochondrial genomes show a very strong bias in nucleotide composition (A + T% > G + C%), which is typical of insect mitochondrial genomes. The 16 Muscomorpha species we analyzed have PCG A + T contents between 71.10% (*Bactrocera dorsalis*) and 78.97% (*Ocyptamus sativus*); The mitochondrial genomes of *Episyrphus* and *Eupeodes* have A + T contents of 78.85% and 78.83%, respectively (Table 4).

The PCG sequence AT-skews of the 16 Muscomorpha species are primarily negative (except in *Episyrphus, O. sativus, Simosyrphus grandicornisand,* and *Haematobia irritans*). All GC-skews are positive, indicating that the PCGs contain a higher percentage of T and C than A and G nucleotides (Table 4), as reported with most other insects^32^.

### Protein-coding genes, codon usage and nucleotide diversity

Nine of the 13 mitochondrial PCGs in *Episyrphus* and *Eupeodes* mitochondrial genomes are located on the J-strand; the other four PCGs are located on the N-strand (Tables 1 and 2). Total PCG length in *Episyrphus* is 11,220 bp, while *Eupeodes* has 11,211 bp of PCG.

All *Episyrphus* and *Eupeodes* mitochondrial genome PCGs start with ATN codons. One, six, and six of the PCGs start with ATA, ATG, and ATT, respectively. Orthologs from the two species have the same start codons. Most PCG stop codons are the canonical TAA, except for *nad5* in *Eupeodes*, which uses an incomplete TA.

**Figure 1:**
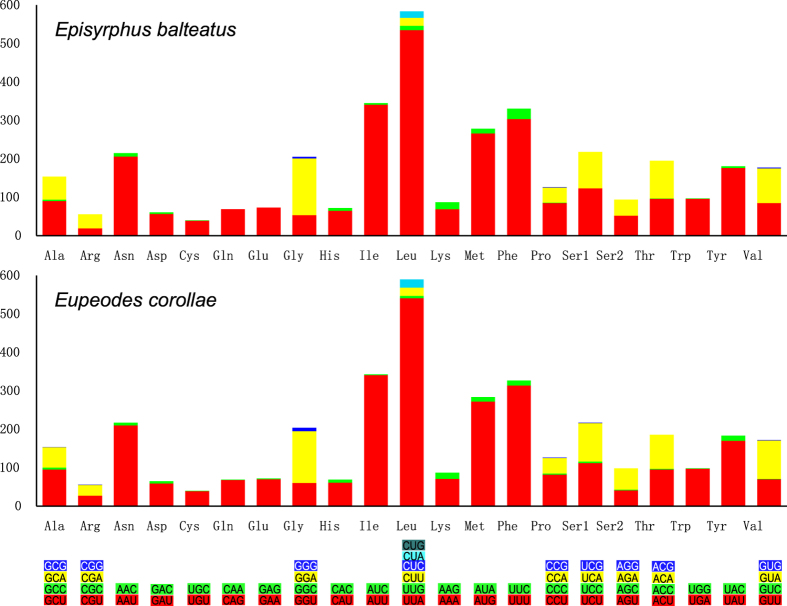
Relative synonymous codon usage (RSCU) of mitochondrial genomes in the two newly sequenced mitochondrial genomes of *Episyrphus balteatus* and *Eupeodes corollae*. The stop codon is not given.

Mitochondrial genome codon usage in *Episyrphus* and *Eupeodes* show a significant bias towards A and T (Fig. 1) as in other species of Muscomorpha (Figure S1). In the *Episyrphus* and *Eupeodes* mitochondrial genomes, Leu, Ile, Phe, and Met are the most frequently encoded amino acids, hence TTA (Leu), ATT (Ile), TTT (Phe), and ATA (Met) are the most frequent codons, as is typical of other insect mitochondrial genomes^30^,^33^,^34^. These frequently used codons exclusively consist of A and T, which contribute to the high A + T content seen in most fly mitochondrial genomes (Figure S1). This preferred codon usage is strongly reflected at third positions by high A/T versus G/C frequencies.

**Figure 2:**
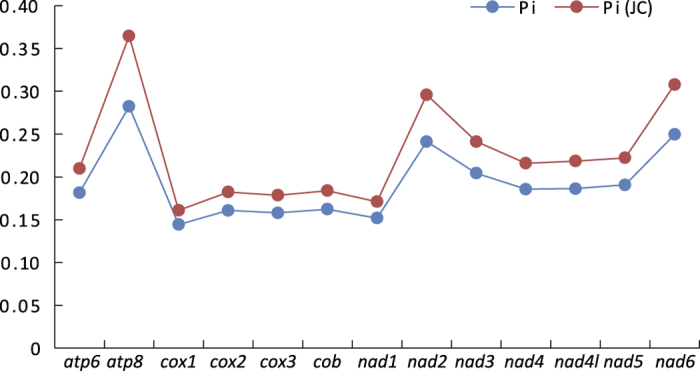
Nucleotide diversity of each protein-coding gene among Muscomorpha. Pi, nucleotide diversity; Pi(JC), Jukes and Cantor-corrected nucleotide diversity. Species used for calculation were listed in Table 1.

Evolutionary rate of protein-coding genes was calculated by using the nucleotide diversity and Jukes and Cantor corrected nucleotide diversity within Muscomorpha. Among the 13 protein-coding genes, five genes of *cox1, cox2, cox3, cob* and *nad1* showed relatively low level, five genes of *atp6, nad6, nad4, nad4l* and *nad5* showed medial level, whereas three genes of *atp8, nad2* and *nad6* showed the highest level of nucleotide diversity (Fig. 2). Relative evolutionary rate among the 13 protein-coding genes in Muscomorpha was similar to previous studies of insect mitochondrial genomes^35^,^36^.

### Transfer RNA and ribosomal RNA genes

All tRNA anticodons from the two newly sequenced mitochondrial genomes are identical to other Muscomorpha species (Tables 1 and 2). Of the 22 tRNA genes total, 14 are located on the J-strand, with the rest on the N-strand.

The *Episyrphus* mitochondrial genome contains 1,477 bp within tRNA genes, at an A + T content of 80.37%. Individual tRNAs range in length from 64 bp (*trnR*) to 72 bp (*trnV*). The *Eupeodes* mitochondrial genome contains 1,479 bp within tRNA genes, at an A + T content of 80.12%. Individual tRNAs range in length from 64 bp (*trnR*) to 72 bp (*trnV*).

**Figure 3:**
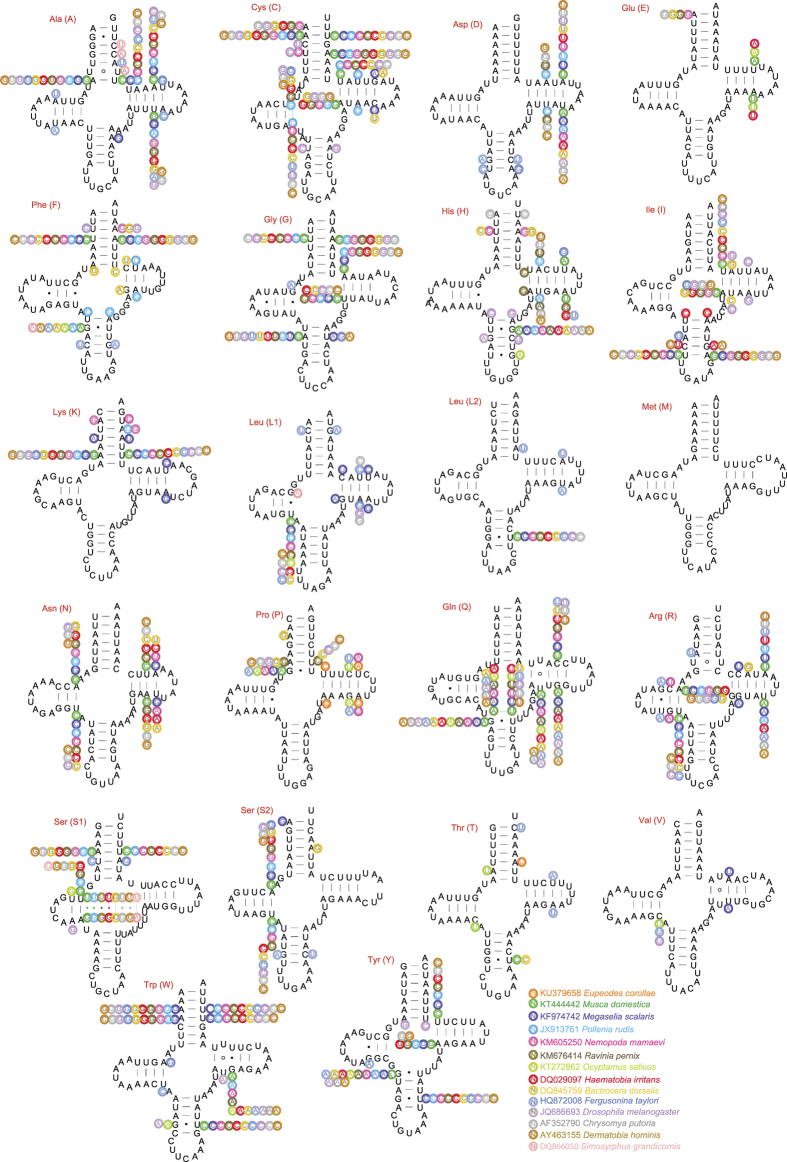
Comparison on the secondary structure of the tRNA genes in Muscomorpha mitochondrial genomes. The secondary structures were draw from tRNA genes of *Episyrphus balteatus*. Variations of each sites in other 14 species of Muscomorpha were indicated near corresponding nucleotide. Each species was marked by a unique color as shown on the right bottom of the figure.

Secondary structure models of the tRNA genes in the two newly sequenced mitochondrial genomes were predicted using the Mitos WebServer^37^ (Fig. 3). In *Episyrphus* and *Eupeodes*, all tRNA genes fold into the canonical clover-leaf structure. The dihydrouridine (DHU) arm of all the tRNAs is a large loop, instead of a conserved stem-and-loop structure; however, this is typical of metazoan mitochondrial genomes^38^. The amino acid acceptor (AA) stem and the anticodon (AC) loop are conserved at 7 bp in all of our tRNA genes. The size of the variable- and D-loop often determine overall tRNA length^39^. The DHU arms in our tRNAs are 2 to 4 bp long, the AC arms are 4 to 5 bp long, and the TΨC arms vary in length from 3 to 5 bp. The variable loops are less consistent, ranging from 4 to 8 bp.

We also compared the variation of stem regions of tRNA genes among 15 species of Muscomorpha. Among the 22 tRNA genes, *trnM* was the most conserved one without any nucleotide variation in stem regions, followed by *trnV* and *trnE* with three site mutations. The *trnC* showed the highest number of site mutation on stem regions (17 sites), followed by the *trnH* (16 sites) (Fig. 2).

Base pairs other than canonical A-Us and C-Gs are occasionally used in our tRNAs, based on predicted tRNA secondary structures. We found six and five mismatched base pairs in the tRNAs from *Episyrphus* and *Eupeodes*, respectively. Among the six mismatched base pairs in *Episyrphus*, five are U-U pairs, located in the AA and TΨC stems; the other is an A-A pair, located in the A-A stem. *Eupeodes* has four U-U pairs, located in the AA and TΨC stems, and an A-A pair, located in the A-A stem.

The two ribosomal RNA genes in the mitochondrial genome, *rrnL* and *rrnS*, are 1,338 bp long, with an A + T content of 84.61%; and 804 bp long, with an A + T content of 83.96%, respectively, in *Episyrphus*. In *Eupeodes, rrnL* is 1334 bp long, with an A + T content of 84.78%; *rrnS* is 795 bp long, with an A + T content of 83.14% (Table 4).

### Phylogenetic relationships

**Figure 4:**
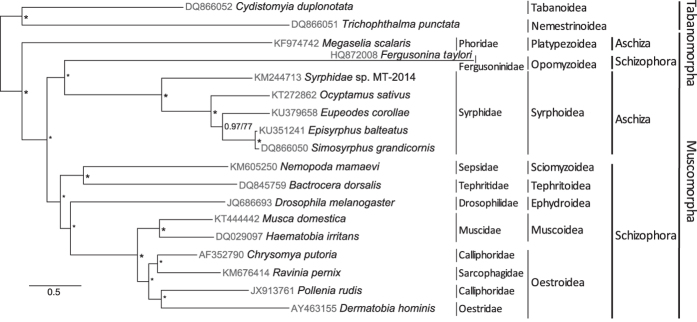
Phylogeny of Muscomorpha inferred from coding nucleotide sequences of the mitochondrial genome (13 PCGs, 22 tRNAs, and 2 rRNA genes), using the Bayesian and maximum likelihood methods. Numbers separated by “/” indicate the posterior probability and bootstrap values of the corresponding nodes (BI / ML). “*” indicate that the node was fully supported by both inferences (1/100).

We reconstructed phylogenies within the Muscomorpha using the nucleotide sequences of the 37 mitochondrial genes. Bayesian and maximum likelihood (ML) methods estimated congruent topologies (Fig. 4). Our analyses supported the monophyly of all superfamilies used in the study. The Aschiza (lower Cyclorrhapha) was found to be a paraphilic group^40^. We included two superfamilies of Platypezoidea and Syrphoidea from Aschiza. Platypezoidea was sister to all other species of Muscomorpha, which is congruent with previous studies^11^,^41^. The five genera of Syrphidae (Syrphoidea) clustered as ((unknown Syrphidae sp.) + (*Ocyptamus* + (*Eupeodes* + (*Episyrphus + Simosyrphus*)))). Syrphoidea and Opomyzoidea formed a lineage, and then sister to the other species of Schizophora. The Opomyzoidea was traditionally considered as a superfamily of Schizophora, which was proved to be a monophyletic group^40^. We study showed that Schizophora was interrupted by Opomyzoidea, which might be caused by the long-branch of Opomyzoidea. In Opomyzoidea, we used one species from family Fergusoninidae, in which, all species are gall-forming flies together with *Fergusobia* (Tylenchida: Neotylenchidae) nematodes. The novel life history of the species from Fergusoninidae might affect the evolutionary pattern of their mitochondrial genomes^2^. The long-branch of Opomyzoidea was also found in previous study based on mitochondrial genome sequences^41^. Phylogenetic relationships of other groups included in our analyses, i.e. ((Sciomyzoidea + Tephritoidea) + (Ephydroidea + (Muscoidea + Oestroidea))), were in accord with previous studies^9^,^13^,^14^,^15^,^16^.

## Methods

### Sampling and DNA extraction

The specimens were collected from Sichuan Province, China. Specimens were initially preserved in 100% ethanol in the field when collected, and then stored at −80 °C prior to DNA extraction. Whole genomic DNA was extracted from the legs and thorax of the specimens using a DNeasy tissue kit (Qiagen, Hilden, Germany), following the manufacturer’s protocols.

### PCR amplification and sequencing

Initially we used a previously designed set of universal primers for insect mitochondrial genomes^1^,^42^ to amplify and sequence partial gene segments. Then we designed specific primers based on the sequenced segments to amplify regions that bridged the gaps between different segments (Table S1). PCR cycling consisted of an initial denaturation step at 96 °C for 3 min, followed by 40 cycles of denaturation at 95 °C for 30 s, annealing at 42–53 °C for 30 s, elongation at 60 °C for 1.5 kb/min (depending on the size of target amplicon), and a final elongation step at 60 °C for 10 min. PCR products were evaluated by agarose gel electrophoreses. PCR components were added following the Takara LA Taq protocols. A primer-walking strategy was used for all the amplifications from both strands (Table S1).

### Mitochondrial genome annotation

Mitochondrion DNA sequences were assembled using Lasergene software (DNAStar, Inc., USA, NewYork). The tRNA genes were initially identified using the Mitos WebServer (http://mitos.bioinf.uni-leipzig.de/index.py)^37^. We set the genetic code to “Invertebrate Mito”. Those tRNAs that could not be found using this approach were confirmed by sequence alignment with their homologs from related species. Secondly, protein-coding genes were identified by BLAST searches in GenBank, using other published mitochondrial genomes from Syrphidae^9^,^16^,^28^. Finally, the rRNA genes and control regions were identified by the boundary of the tRNA genes, and by comparison with other insect mitochondrial genomes.

### Comparative analysis of the mitochondrial genomes from Symphyta

We compared the mitochondrial genomes of 16 species from the Muscomorpha, including our two newly sequenced genomes. Gene arrangement, base composition, and PCG codon usage features were analyzed. Because several tRNA genes were not available for some species, we analyzed base composition using only the PCGs. Furthermore, the unknown Syrphidae sp. sequence lacked *nad2* data; therefore, we excluded this species from these analyses.

We calculated base composition using MEGA6^43^. The AT-skew and GC-skew were calculated according to Hassanin, *et al*.^31^: AT-skew = (A% − T%)/(A% + T%) and GC-skew = (G% − C%)/(G% + C%). The intergenic spacers and overlapping regions between genes were counted manually. The relative synonymous codon usage (RSCU) of all protein-coding genes was calculated using CodonW (written by John Peden, University of Nottingham, UK). Nucleotide diversity and Jukes and Cantor-corrected nucleotide diversity were calculated for species of Muscomorpha using DnaSP v5^44^.

### Phylogenetic analysis

We used 14 Muscomorpha species with published mitochondrial genomes, and our two newly sequenced mitochondrial genomes for phylogenetic analyses (Table 1). The 16 species are classified as belonging to two sections, Aschiza and Schizophora. We selected Aschiza sequences belonging to two superfamilies, Platypezoidea and Syrphoidea. Schizophora is classified as two subsections. We selected sequences from both subsections, and selected sequences from six superfamilies within them. *Cydistomyia duplonotata* and *Trichophthalma punctata* (Tabanomorpha: Tabanoidea: Tabanidae) were used as outgroups because of the close relationship between Tabanomorpha and Muscomorpha^11^.

MAFFT version 7.205, which implements consistency-based algorithms, was used for the alignment of protein-coding and RNA genes^45^. We used the G-INS-I and Q-INS-I algorithms in MAFFT^46^ for protein-coding and RNA alignment, respectively. The alignment of the nucleotide sequences was guided by the amino acid sequence alignment using the Perl script TranslatorX version 1.1^47^.

Data partitioning, and the ability to apply specific models to different partitions, is ideal for analyzing mitochondrial genomes^2^. We used PartitionFinder version 1.1.1^48^ to simultaneously confirm partition schemes and choose substitution models for the matrix. The DNA sequence search model was set to “mrbayes”. The greedy algorithm was used, with estimated, linked branch lengths, to search for the best-fit partitioning model.

We constructed phylogenies among the Muscomorpha with the Bayesian inference method (BI) using Mrbayes version 3.2.5^49^, and the ML method using RAxML version 8.0.0^50^. In BI, the GTR + I + G, GTR + G, HKY + I + G, and HKY + G models were used with corresponding partitions (Table S2). Four simultaneous Markov chains were run for 10 million generations, with tree sampling occurring every 1,000 generations, and a burn-in of 25% of the trees. We used the GTR + G model for each ML analysis. We conducted 200 ML runs to find the highest-likelihood tree, then analyzed 1,000 bootstrap replicates.

## Additional Information

**How to cite this article:** Pu, D.-q. *et al*. Mitochondrial genomes of the hoverflies *Episyrphus balteatus* and *Eupeodes corollae* (Diptera: Syrphidae), with a phylogenetic analysis of Muscomorpha. *Sci. Rep.* **7**, 44300; doi: 10.1038/srep44300 (2017).

**Publisher's note:** Springer Nature remains neutral with regard to jurisdictional claims in published maps and institutional affiliations.

## Supplementary Material

Supplemental Information

## Figures and Tables

**Table 1:** Mitochondrial genomes used in this study.

	Sections	Species	Superfamily	Family	Accession number	References
Ingroup	Aschiza	*Episyrphus balteatus*	Syrphoidea	Syrphidae	KU351241	This paper
		*Eupeodes corollae*	Syrphoidea	Syrphidae	KU379658	This paper
		*Ocyptamus sativus*	Syrphoidea	Syrphidae	KT272862	Junqueira, *et al*.^16^
		*Simosyrphus grandicornis*	Syrphoidea	Syrphidae	DQ866050	Cameron, *et al*.^9^
		*Syrphidae* sp.	Syrphoidea	Syrphidae	KM244713	Tang, *et al*.^28^
		*Megaselia scalaris*	Platypezoidea	Phoridae	KF974742	Zhong, *et al*.^51^
	Schizophora	*Bactrocera dorsalis*	Tephritoidea	Tephritidae	DQ845759	Zhong, *et al*.^51^
		*Drosophila melanogaster*	Ephydroidea	Drosophilidae	JQ686693	Chen, *et al*.^52^
		*Fergusonina taylori*	Opomyzoidea	Fergusoninidae	HQ872008	Nelson, *et al*.^53^
		*Nemopoda mamaevi*	Sciomyzoidea	Sepsidae	KM605250	Li, *et al*.^11^
		*Haematobia irritans*	Muscoidea	Muscidae	DQ029097	Unpublished
		*Musca domestica*	Muscoidea	Muscidae	KT444442	Unpublished
		*Chrysomya putoria*	Oestroidea	Calliphoridae	AF352790	Junqueira, *et al*.^54^
		*Dermatobia hominis*	Oestroidea	Oestridae	AY463155	Unpublished
		*Pollenia rudis*	Oestroidea	Calliphoridae	JX913761	Nelson, *et al*.^55^
		*Ravinia pernix*	Oestroidea	Sarcophagidae	KM676414	Unpublished
Outgroup		*Cydistomyia duplonotata*	Tabanoidea	Tabanidae	DQ866052	Cameron, *et al*.^9^
		*Trichophthalma punctata*	Nemestrinoidea	Nemestrinidae	DQ866051	Cameron, *et al*.^9^

**Table 2:** Annotation of the *Episyrphus balteatus* mitochondrial genome.

Gene	Position (bp)	Strand	Length (bp)	Intergenic nucleotides	Anti or start/stop codon
*trnI*	1–66	+	66	2	GAT
*trnQ*	69–137	−	69	14	TTG
*trnM*	152–220	+	69	0	CAT
*nad2*	221–1252	+	1032	3	ATT/TAA
*trnW*	1256–1323	+	68	8	TCA
*trnC*	1332–1398	−	67	14	GCA
*trnY*	1413–1478	−	66	5	GTA
*cox1*	1484–3043	+	1560	−5	ATT/TAA
*trnL2*	3039–3104	+	66	2	TAA
*cox2*	3107–3790	+	684	5	ATG/TAA
*trnK*	3796–3866	+	71	60	CTT
*trnD*	3927–3993	+	67	0	GTC
*atp8*	3994–4155	+	162	−7	ATT/TAA
*atp6*	4149–4826	+	678	35	ATG/TAA
*cox3*	4862–5650	+	789	3	ATG/TAA
*trnG*	5654–5719	+	66	0	TCC
*nad3*	5720–6073	+	354	4	ATT/TAA
*trnA*	6078–6146	+	69	−1	TGC
*trnR*	6146–6209	+	64	2	TCG
*trnN*	6212–6278	+	67	−1	GTT
*trnS1*	6278–6344	+	67	1	GCT
*trnE*	6346–6410	+	65	23	TTC
*trnF*	6434–6500	−	67	−1	GAA
*nad5*	6500–8221	−	1722	15	ATT/TAA
*trnH*	8237–8303	−	67	−1	GTG
*nad4*	8303–9643	−	1341	−7	ATG/TAA
*nad4l*	9637–9933	−	297	2	ATG/TAA
*trnT*	9936–10000	+	65	0	TGT
*trnP*	10001–10066	−	66	2	TGG
*nad6*	10069–10593	+	525	3	ATT/TAA
*cob*	10597–11733	+	1137	1	ATG/TAA
*trnS2*	11735–11802	+	68	16	TGA
*nad1*	11819–12757	−	939	10	ATA/TAA
*trnL1*	12768–12832	−	65	0	TAG
*rrnL*	12833–14170	−	1338	0	
*trnV*	14171–14242	−	72	0	TAC
*rrnS*	14243–15046	−	804	0	
A+T-rich region	15047–16175	−	1129	0	

+ majority strand; − minority strand; negative intergenic nucleotides indicate overlapping sequences between adjacent genes.

**Table 3:** Annotation of the *Eupeodes corollae* mitochondrial genome.

Gene	Position (bp)	Strand	Length (bp)	Intergenic nucleotides	Anti or start/stop codon
*trnI*	1–66	+	66	−3	GAT
*trnQ*	64–133	−	70	7	TTG
*trnM*	141–209	+	69	0	CAT
*nad2*	210–1238	+	1029	−2	ATT/TAA
*trnW*	1237–1304	+	68	4	TCA
*trnC*	1309–1374	−	66	10	GCA
*trnY*	1385–1450	−	66	22	GTA
*cox1*	1473–3029	+	1557	−5	ATT/TAA
*trnL2*	3025–3090	+	66	2	TAA
*cox2*	3093–3776	+	684	0	ATG/TAA
*trnK*	3777–3847	+	71	47	CTT
*trnD*	3895–3961	+	67	0	GTC
*atp8*	3962–4123	+	162	−7	ATT/TAA
*atp6*	4117–4794	+	678	16	ATG/TAA
*cox3*	4811–5599	+	789	3	ATG/TAA
*trnG*	5603–5669	+	67	0	TCC
*nad3*	5670–6023	+	354	3	ATT/TAA
*trnA*	6027–6095	+	69	−1	TGC
*trnR*	6095–6158	+	64	13	TCG
*trnN*	6172–6238	+	67	−1	GTT
*trnS1*	6238–6304	+	67	5	GCT
*trnE*	6310–6376	+	67	26	TTC
*trnF*	6403–6469	−	67	−1	GAA
*nad5*	6469–8189	−	1721	15	ATT/TA
*trnH*	8205–8270	−	66	−1	GTG
*nad4*	8270–9610	−	1341	−7	ATG/TAA
*nad4l*	9604–9900	−	297	2	ATG/TAA
*trnT*	9903–9967	+	65	0	TGT
*trnP*	9968–10033	−	66	2	TGG
*nad6*	10036–10560	+	525	3	ATT/TAA
*cob*	10564–11700	+	1137	7	ATG/TAA
*trnS2*	11708–11775	+	68	17	TGA
*nad1*	11792–12730	−	939	10	ATA/TAG
*trnL1*	12741–12805	−	65	0	TAG
*rrnL*	12806–14139	−	1334	0	
*trnV*	14140–14211	−	72	0	TAC
*rrnS*	14212–15006	−	795	0	
A+T-rich region	15007–15326	−			

Symbols are as in Table 2.

**Table 4:** Nucleotide composition in regions of Muscomorpha mitochondrial genomes.

Species	Accession number	PCGs								*rrnl*		*rrns*	
		T	C	A	G	Length (bp)	AT (%)	AT-skew	GC-skew	Length (bp)	AT (%)	Length (bp)	AT (%)
Episyrphus balteatus	KU351241	39.90	11.66	38.95	9.49	11220	78.85	−0.0121	−0.1024	1338	84.60	804	83.96
Eupeodes corollae	KU379658	39.25	11.66	39.59	9.51	11211	78.83	0.0043	−0.1016	1334	84.78	795	83.14
Ocyptamus sativus	KT272862	39.71	11.68	39.26	9.35	11175	78.97	−0.0058	−0.1106	1314	84.40	778	82.78
Simosyrphus grandicornis	DQ866050	39.75	11.81	39.09	9.35	11208	78.84	−0.0084	−0.1164	1339	84.99	804	83.83
Megaselia scalaris	KF974742	37.26	14.81	37.66	10.26	11301	74.92	0.0053	−0.1814	1318	81.03	786	79.64
Bactrocera dorsalis	DQ845759	32.94	17.85	38.16	11.05	11175	71.10	0.0734	−0.2353	1333	79.52	790	74.94
Drosophila melanogaster	JQ686693	38.16	12.78	39.06	10.00	11169	77.22	0.0117	−0.1219	1323	82.84	786	80.15
Fergusonina taylori	HQ872008	36.13	14.19	39.92	9.76	11160	76.05	0.0498	−0.1852	1306	82.54	780	82.05
Nemopoda mamaevi	KM605250	35.84	16.28	36.88	11.00	11181	72.72	0.0144	−0.1934	1321	80.62	783	77.65
Haematobia irritans	DQ029097	39.04	12.29	38.63	10.04	11178	77.67	−0.0053	−0.1010	1321	82.66	784	78.83
Musca domestica	KT444442	37.84	13.61	38.24	10.31	11184	76.08	0.0053	−0.1379	1326	81.00	783	78.29
Chrysomya putoria	AF352790	36.74	14.59	38.23	10.44	11187	74.97	0.0199	−0.1657	1329	81.79	785	76.94
Dermatobia hominis	AY463155	35.96	15.08	39.31	9.65	11187	75.27	0.0445	−0.2198	1324	83.08	788	78.68
Pollenia rudis	JX913761	37.29	14.14	38.52	10.04	11172	75.81	0.0163	−0.1695	1328	81.63	448	74.11
Ravinia pernix	KM676414	36.88	14.33	38.63	10.17	11181	75.50	0.0232	−0.1698	1328	82.08	786	77.48
