# Supplementary material for: Mitochondrial genomes of the hoverflies Episyrphus balteatus and Eupeodes corollae (Diptera: Syrphidae), with a phylogenetic analysis of Muscomorpha
Source: Sci Rep. 2017 Mar 9;7:44300. doi: 10.1038/srep44300 (PMC5343577; doi:10.1038/srep44300)
Supplement: Supplemental Information [file srep44300-s1.doc]

Mitochondrial genomes of the hoverflies *Episyrphus* *balteatus* and *Eupeodes* *corollae* (Diptera: Syrphidae), with a phylogenetic analysis of Muscomorpha

De-qiang Pu^1^, Hong-ling Liu^2^, Yi-yun Gong^1^, Pei-cheng Ji^1^, Yue-jian Li^3^, Fang-sheng Mou^1^, Shu-jun Wei^4^

^1^ Industrial Crop Research Institute, Sichuan Academy of Agricultural Sciences, Chengdu 610300, China

^2^ Institute of Plant Protection, Sichuan Academy of Agricultural Sciences, Chengdu 610066, China

^3^ Institute of Horticulture Research, Sichuan Academy of Agricultural Sciences, Chengdu 610066, China

^4^ Institute of Plant and Environmental Protection, Beijing Academy of Agriculture and Forestry Sciences, Beijing 100097, China

**Table S1** Primers used for amplification and sequencing of the mitochondrial genomes from *Eupeodes corollae* and *Episyrphus balteatus*

|  | **Region** | **Primer position** | | **Primer name** | **Primer pairs** |
| --- | --- | --- | --- | --- | --- |
| *Episyrphus balteatus* | *TI-nad2* | 34-339 | | TI-J34 | GCCTGATAAAAAGGRTTAYYTTGATA^a^ |
|  |  |  |  | Eb-339-R | CTAATCCTATTCAAGCACCT^b^ |
|  | *TM-cox1* | 210-2195 | | TM-J210 | AATTAAGCTACTAGGTTCATACCC^a^ |
|  |  |  |  | EB-2195-R3 | TAAGCTCGAGTATCTACGTCTA^b^ |
|  |  |  |  | T1029009 | CAAAAGCATGGGCTGTAACA^c^ |
|  | *cox1-cox2* | 2195-3665 | | C1-J2195 | TGATTCTTTGGWCACCCWGAAGT^a^ |
|  |  |  |  | C2-N3665 | CCACAAATTTCTGAACATTGa |
|  | *cox2-cox3* | 3400-3665 | | C2-J-3400 | ATTGGACATCAATGATATTGA^a^ |
|  |  |  |  | C2-N3665 | CCACAAATTTCTGAACATTG^a^ |
|  | *cox2-cox3* | 3665-4292 | | Eb-3665-4792-F3 | TCTTGAACAGTTCCTGCTTTGG^b^ |
|  |  |  |  | Eb-3665-4792-R3 | ATTCCTCATCGTAAACCTGTTG^b^ |
|  |  |  |  | T1020002 | TTCCCTTTTGATTAGGATTACC^c^ |
|  | *cox3-nad3* | 4292-5731 | | C3-J4792 | GTTGATTATAGACCWTGRCCa |
|  |  |  |  | N3-N5731 | TTAGGGTCAAATCCRCAYTCa |
|  | *nad3-nad5* | 5731-7077 | | EB-5731-7077-F4 | AAGCAGCAGCATGATATTGAC^b^ |
|  |  |  |  | EB-5731-7077-R4 | ATGTGCTGGTGCTATTATTC^b^ |
|  | *nad5-nad5* | 7077-7793 | | N5-J7077 | TTAAATCCTTWGARTAAAAYCC^a^ |
|  |  |  |  | N5-N7793 | TTAGGTTGRGATGGNYTAGG^a^ |
|  | *nad5-nad4* | 7793-8727 | | EB-7793-11876-F2 | AAAATACATCCCCAACTCG^b^ |
|  |  |  |  | N4-N8727 | AAATCTTTRATTGCTTATTCWTC^a^ |
|  | *nad4-nad4* | 8641-9753 | | N4-J8641-EB | AAACCTGATGAACATAATCCATG^b^ |
|  |  |  |  | N4-N9153 | TGAGGTTATCAACCNGARCGa |
|  | *nad4-cob* | 8944-11010 | | N4-J-8944 | GGAGCTTCAACATGAGCTTT^a^ |
|  |  |  |  | CB-N11010-EC | TATCTACTGCAAATCCTCCTCA^b^ |
|  |  |  |  | T1101017 | GAAACCAAAACTGATAACCC^c^ |
|  | *cob-nad1* | 10933-12067 | | CB-J10933 | GTTCTACCTTGAGGNCAAATRTC^a^ |
|  |  |  |  | N1-N12067-Eb | AATCGAACACCATTTGATTTTGC^b^ |
|  | *nad1-lr* | 11876-13000 | | N1-J11876 | CGAGGTAAAGTMCCWCGAACYCA^a^ |
|  |  |  |  | LR-N13000 | TTACCTTAGGGATAACAGCGTAA^a^ |
|  | *lr-sr* | 12888-14220 | | LR-J12888-Eb | CCGGTTTGAACTCAGATCATGTAA^a^ |
|  |  |  |  | SR-N14220-Eb | GAGTGACGGGCGATATGTACATAT^b^ |
|  | *lr-sr* | 13900-14745 | | LR-J13900 | TTTGATAAACYCTGATACAMAAGa |
|  |  |  |  | SR-N14745 | GTGCCAGCAGYYGCGGTTANAC^a^ |
|  | *sr-nad2* | 14610-15159 | | SR-J14610 | ATAATAGGGTATCTAATCCTAGT^a^ |
|  |  |  |  | Eb-339-R | CTAATCCTATTCAAGCACCT^b^ |
|  |  |  |  | T1109010 | GATTTAAATTGTAAAGGG^c^ |
|  |  |  |  | EB-15159-F | CTACCCCTAATAATAAAT^c^ |
|  | *sr-tm* | 15082-200 | | EB-15082-F1 | AATTTTGAGTAACTGATCTA^b^ |
|  |  |  |  | TM-N200 | ACCTTTATAARTGGGGTATGARCC^a^ |
| *Eupeodes corollae* | *tI-cox1* | | 34-2353 | TI-J34 | GCCTGATAAAAAGGRTTAYYTTGATA^a^ |
|  |  | |  | C1-N2353 | GCTCGTGTATCAACGTCTATWCC^a^ |
|  |  | |  | T1101018 | GGAGGATTAAATCAAACATC^c^ |
|  | *tw-cox1* | | 1301-2353 | TW-J1301 | GTTAAWTAAACTAATARCCTTCAAA^a^ |
|  |  | |  | C1-N2353 | GCTCGTGTATCAACGTCTATWCC^a^ |
|  | *cox1-cox2* | | 2195-3665 | C1-J2195 | TGGTCACCCTGAAGTTTAT^a^ |
|  |  | |  | C2-N3665 | CCACAAATTTCTGAACATTG^a^ |
|  | *cox2-cox3* | | 3400-4908 | C2-J-3400-EC | AATTGGTCATCAATGATACTGA^b^ |
|  |  | |  | C3-N4908-Eb | CGTGATACATCTCGTCATCATTG^b^ |
|  | *cox3-nad3* | | 4792-5731 | C3-J4792 | GTTGATTATAGACCWTGRCC^a^ |
|  |  | |  | N3-N5731 | TTAGGGTCAAATCCRCAYTC^a^ |
|  | *nad3-nad5* | | 5731-7211 | Eb-5731-7077-F4 | AAGCAGCAGCATGATATTGAC^b^ |
|  |  | |  | N5-N7211-EC | TTAAAGCTTTATTATTTATATGT^b^ |
|  | *nad5-nad5* | | 7077-7793 | N5-J7077 | TTAAATCCTTWGARTAAAAYCC^a^ |
|  |  | |  | N5-N7793 | TTAGGTTGRGATGGNYTAGG^a^ |
|  | *nad5-nad4* | | 7572-9629 | N5-J7572-EC | AAAAGGAATTTGAGCACTTTTA^b^ |
|  |  | |  | N4L-N9629 | GTTTGTGAGGGWGYTTTRGG^a^ |
|  |  | | 8641-9153 | N4-J8641-EC | CCTGAAGAACATAATCCATG^b^ |
|  |  | |  | N4-N9153-EC | TGGGGTTATCAACCTGAACG^b^ |
|  | *nad4-cob* | | J8944-11010 | N4-J8944 | GGAGCTTCAACATGAGCTTT^a^ |
|  |  | |  | CB-N11010-EC | TATCTACTGCAAATCCTCCTCA^b^ |
|  | *cob-cob* | | 10933-11335 | CB-J10933 | GTTCTACCTTGAGGNCAAATRTC^a^ |
|  |  | |  | EC-7793-11335-R | CTTCTACTGGACGAGCT^b^ |
|  | *cob-lr* | | 11335-13000 | CB-J11335 | CATATTCAACCWGAATGRTA^a^ |
|  |  | |  | EC-7793-11335-R | TTACCTTAGGGATAACAGCGTAA^b^ |
|  | *lr-sr* | | 12888-14745 | LR-J12888-EC | CCGGTTTGAACTCAGATCATGTA^b^ |
|  |  | |  | SR-N14745 | GTGCCAGCAGYYGCGGTTANAC^a^ |
|  | *sr-tm* | | 14563-200 | EC-14563-F1 | TAAATAATAGGGTATCTAATCC^b^ |
|  |  | |  | TM-N200 | ACCTTTATAARTGGGGTATGARCC^a^ |
|  | *sr-nad2* | | 14610-993 | SR-J14610 | ATAATAGGGTATCTAATCCTAGT^a^ |
|  |  | |  | N2-N993 | GGTAAAAATCCTAAAAATGGNGG^a^ |

^a^ Primers from Simon et al., 2006.

^b^ Primers designed in this study.

^C^ Walking Primers.

**Table S2** The best partition scheme and substitution models used for phylogenetic analysis.

| **Partitions** | **Models** | **Partition** |
| --- | --- | --- |
| 1 | GTR+I+G | *c1p1, c2p1, c3p1, cbp1* |
| 2 | GTR+I+G | *a6p1, n3p1, trnG, trnI, trnK, trnL1, trnL2, trnN, trnP, trnQ, trnS1, trnS2, trnV, trnW* |
| 3 | GTR+I+G | *a8p1, n2p1, n6p1* |
| 4 | GTR+I+G | *rrnl, rrns, trnH, trnR, trnT* |
| 5 | GTR+I+G | *a6p2, c1p2, c2p2, c3p2, cbp2,n3p2, trnM* |
| 6 | GTR+I+G | *n1p2, n4lp2, n4p2, n5p2* |
| 7 | GTR+I+G | *n1p1, n4lp1, n4p1, n5p1, trnC, trnF, trnY* |
| 8 | GTR+G | *a8p2, n2p2, n6p2* |
| 9 | HKY+I+G | *trnA, trnD, trnE* |
| 10 | GTR+I+G | *a6p3, a8p3, c1p3, c2p3, c3p3,cbp3, n2p3, n3p3, n6p3* |
| 11 | HKY+I+G | *n1p3, n4lp3, n4p3, n5p3* |

The data are partitioned by gene and codon of protein-coding genes. For protein-coding genes, partitions are named by abbreviated two-letters gene name and position of partition. P1, p2 and p3 after name of protein-coding gene indicate the first, second and third codon position of corresponding gene.


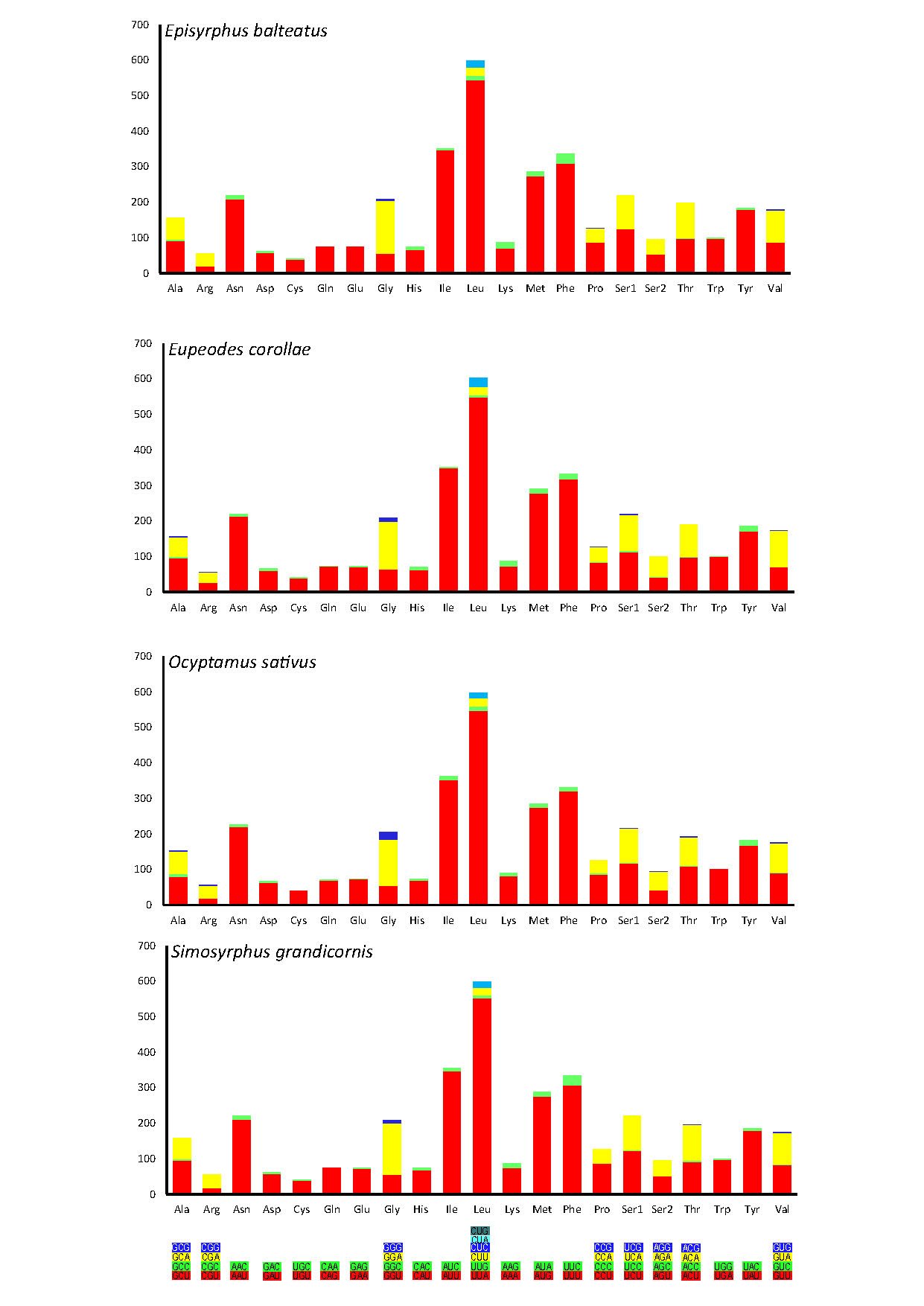


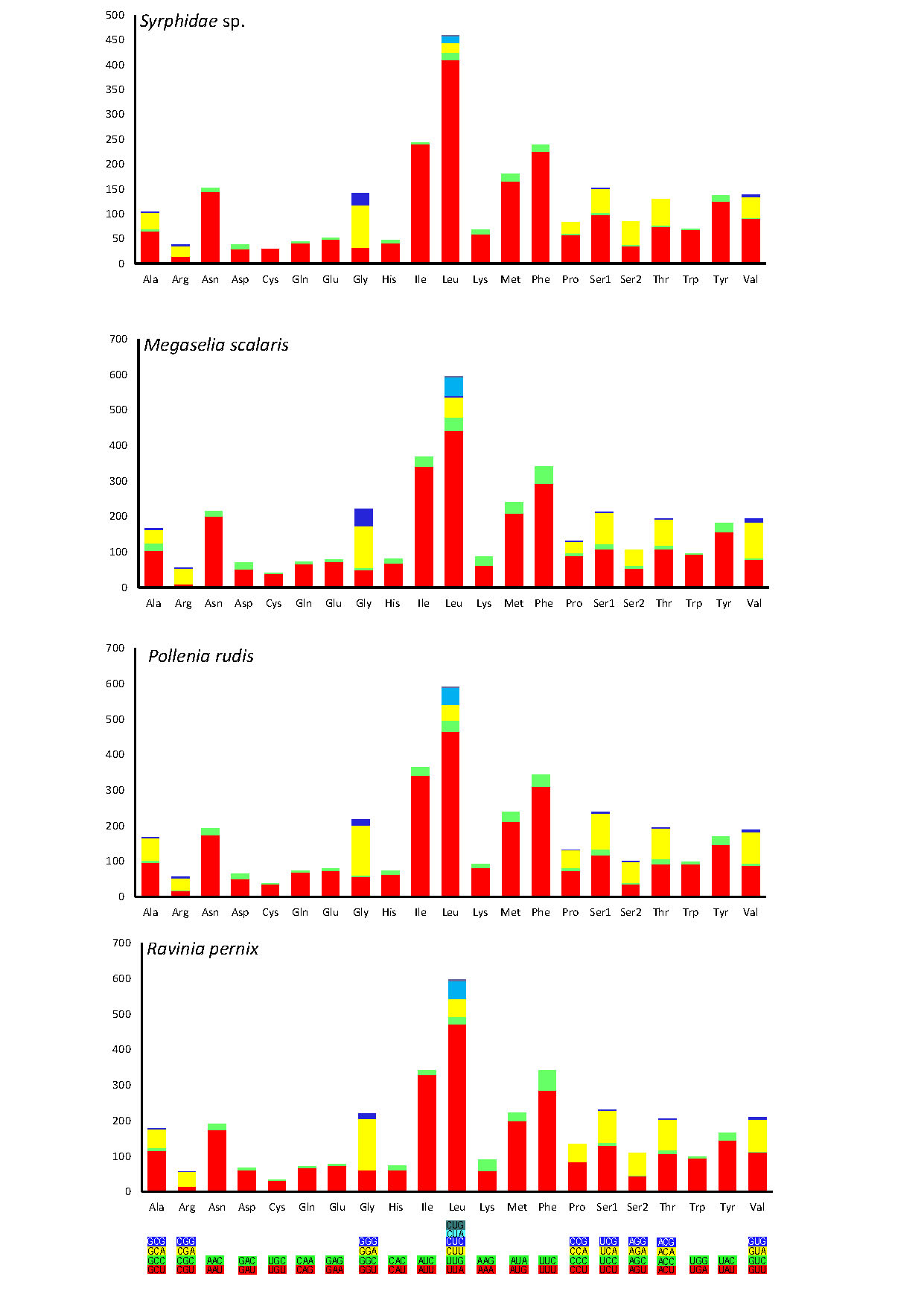


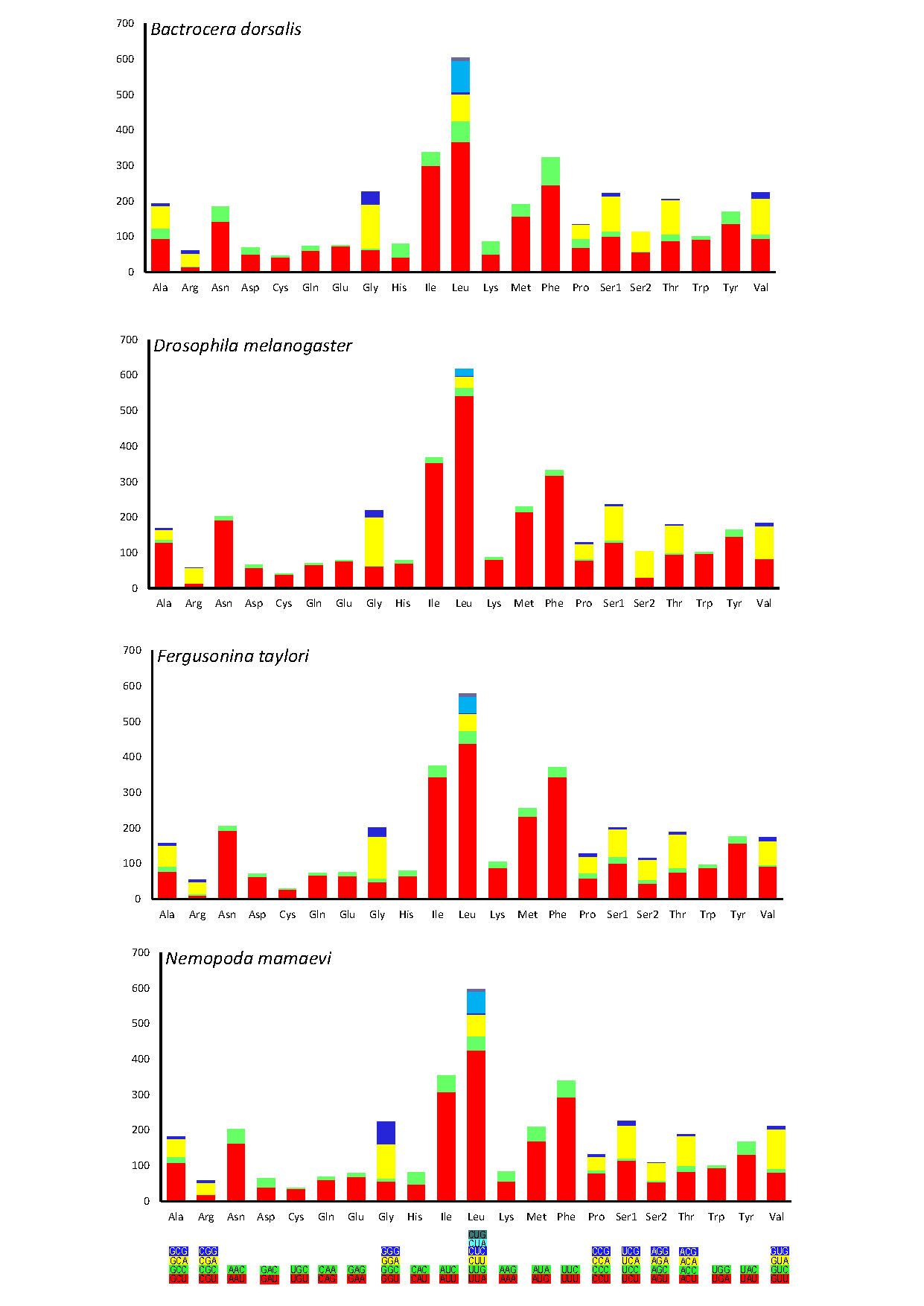


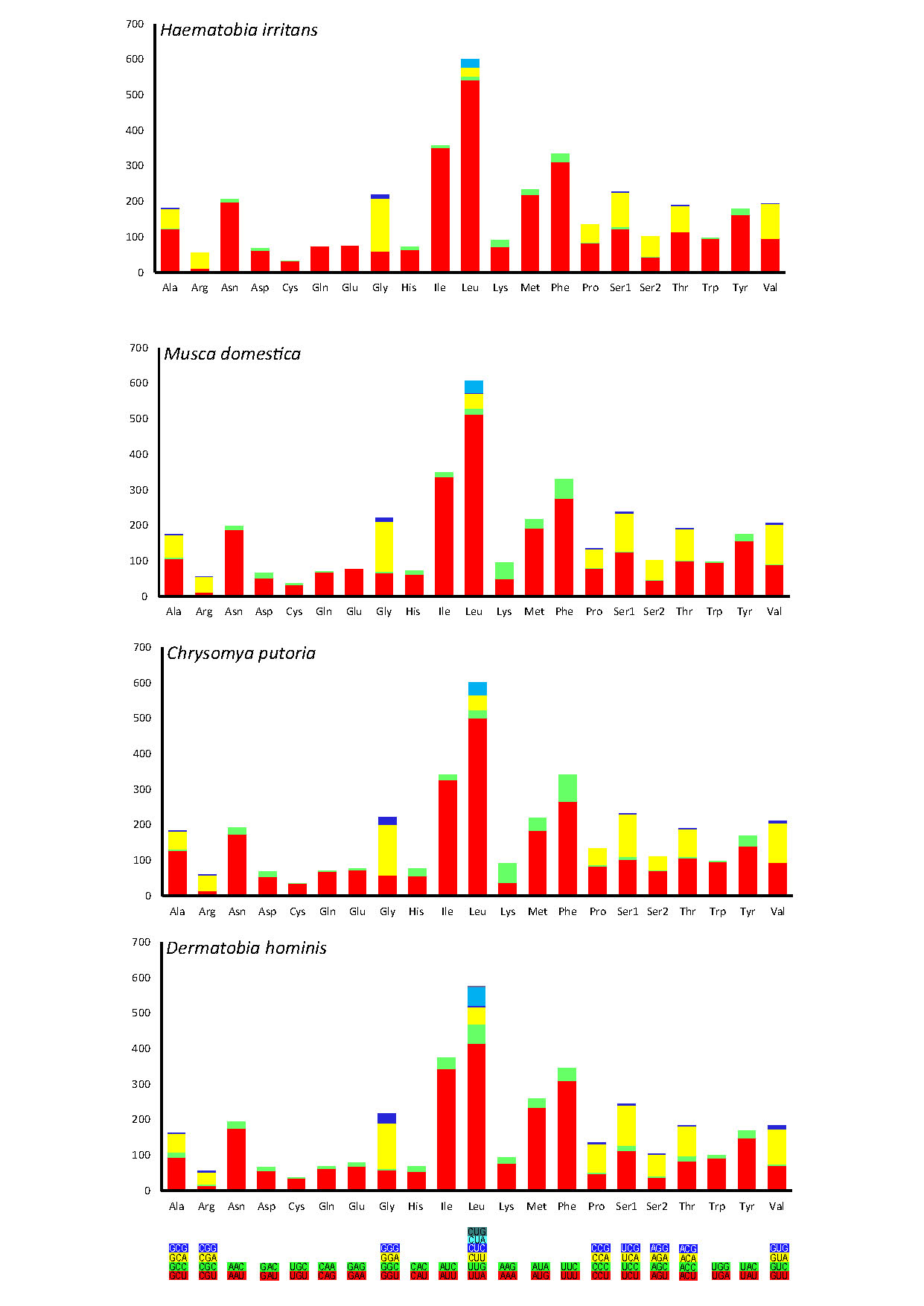


**Figure S1** Relative synonymous codon usage (RSCU) of mitochondrial genomes in [Muscomorpha](https://en.wikipedia.org/wiki/Muscomorpha). The stop codon is not given.
